# Listening to the Shepard-Risset Glissando: the Relationship between Emotional Response, Disruption of Equilibrium, and Personality

**DOI:** 10.3389/fpsyg.2016.00300

**Published:** 2016-03-04

**Authors:** Eveline Vernooij, Angelo Orcalli, Franco Fabbro, Cristiano Crescentini

**Affiliations:** ^1^Music and Audio Research Laboratories, Department of Languages and Literature, Communication, Education and Society, University of UdineUdine, Italy; ^2^IRCCS “Eugenio Medea”San Vito al Tagliamento, Italy; ^3^Department of Medical and Biological Sciences, University of UdineUdine, Italy

**Keywords:** music, emotion, personality, auditory illusions, Shepard-Risset glissando, equilibrium

## Abstract

The endless scale illusion, obtained by cyclically repeating a chromatic scale made up of Shepard tones, has been used in a variety of musical works. Music psychology and neuroscience has been interested in this particular psychoacoustic phenomenon mainly for studying the cognitive processes of pitch perception involved. In the present study, we investigated the emotional states induced by the Shepard-Risset glissando, a variant of the Shepard scale. For this purpose we chose three musical stimuli: a Matlab-generated Shepard Risset glissando, Jean-Claude Risset's *Computer Suite from Little Boy*, which presents a Shepard-Risset glissando integrated in the aesthetic context of a composition, and an ordinary orchestral glissando taken from the opening of Iannis Xenakis's *Metastasis*. Seventy-three volunteers completed a listening experiment during which they rated their emotional response to these stimuli on a seven-point Likert scale and indicated whether they had experienced a disruption of equilibrium. Personality was also measured with the Five-Factor Model of personality traits. The results show that negative emotions were most strongly evoked during listening to each of the stimuli. We also found that the Shepard-Risset glissando illusion, both within the aesthetic context of a musical composition and on its own, was capable of evoking disruption of equilibrium, frequently leading to the associated feeling of falling. Moreover, generally for the Shepard-Risset glissando illusion, higher negative emotional ratings were given by individuals who had experienced a feeling of disturbance of equilibrium relative to those who had not had this experience. Finally, we found a complex pattern of relationships between personality and the subjective experience of the glissando. Openness to experience correlated positively with positive emotion ratings for the Computer Suite, while agreeableness correlated negatively with positive emotion ratings for the Matlab stimulus. Moreover, results indicated higher (Bonferroni-uncorrected) neuroticism for those who experienced an equilibrium disturbance relative to subjects who did not have this experience during listening to the Computer Suite. These findings suggest that musical paradoxes may be of interest not only for the insights they provide on our perceptual system, but also for the richness of the emotional experience elicited during listening.

## Introduction

The digital resources of synthesis initially developed for artistically reasons have contributed significantly to our understanding of the perception of musical sound. Vice versa, the study and exploitation of cognitive principles and effects have made their way into the realm of musical composition. Thanks to the flexibility of programming, the physical parameters of acoustic sounds can be controlled directly and sounds can be manufactured with unprecedented reproducibility and precision. However, a purely mathematical approach to sound synthesis is not enough to manufacture artistically satisfying sounds, instead one has to take into account the relation between the physical structure—which the composer controls when he specifies the synthesis data—and the aural effect—which determines the musical impact (Risset, 1988).

One of the most intriguing psychoacoustic effects employed in musical composition is that of auditory illusions. Illusions contain ambiguous images that allow for multiple distinct interpretations or that can lure the brain into making perceptual errors (Kandel, 2012, p. 207). Ernst Gombrich made an art historian's case long ago for an inextricable connection between the two phenomena named in the title of his singularly perceptive book *Art and illusion* (Gombrich, 1960). Similarly, Shepard (1990) argues that ambiguity is the principal source of the inexhaustible richness of art.

One of the best known auditory illusions is the Shepard scale, also known as the endless scale illusion. The Shepard scale consists of a sequence of auditory figures that are composed of patterns which, when played in a continuous loop, give the impression of rising or descending infinitely in pitch. This illusion is reminiscent of the Penrose staircase, made popular by M. C. Escher's lithograph *Ascending and Descending*. The endless scale was originally designed by Shepard (1964) and has come to be known as the Shepard scale. Shepard used a set of 12 computer-generated complex tones a semitone apart, each consisting of octave-separated sinusoidal components whose amplitudes are shaped by a stationary Gaussian envelope. Pitch height (i.e., the height of the octave in which the note should be placed) of these tones is ambiguous, because of the absence of a complete harmonic series. As a consequence, when these tones are played as a cyclically repeated chromatic scale, the impression is that of an infinite sequence that progresses monotonically in pitch. Thus, the Shepard scale creates an illusion because it contains ambiguous tones that lure the brain into making perceptual errors. Although the stimulus consists of short, continuously repeated patterns, the listener perceives a single pattern that progresses endlessly in pitch. The auditory effect is therefore paradoxical: since each tone is perceived as being clearly lower (or higher, depending on the direction of the scale or glissando) than the preceding one, the Shepard Scale appears to descend (or ascend, depending on the direction of the scale) infinitely, even though this interpretation is not reflected in the physical structure of the stimulus. In 1968 the French composer Jean-Claude Risset (Risset, 1969a) developed a variant of the discrete Shepard scale for continuous gliding tones, known as the Shepard-Risset Glissando.

The endless scale illusion, both in its discrete and its continuous version, has been used in twentieth-century musical practice ranging from the classical repertoire to pop/rock songs: from Alban Berg's opera *Wozzeck*, Act III, scene 4 (Berg, 1925/2003, disc 2 track 3)—Shepard scale ante litteram—, Jean-Claude Risset's *Mutations* (Risset, 1969b/2001, track 6), to Pink Floyd's *Echoes* (Pink Floyd, 1971/2011, track 6) and the Beatles' *A day in the life* (The Beatles, 1967/2009, track 13). Particularly striking is the use of endless descending glissandi in Risset's *Computer Suite from Little Boy* (Risset, 1968/1988, track 5). Here the composer endeavors to convey the feeling of the pilot who identifies himself with “Little Boy,” the atomic bomb dropped on Hiroshima. Since the fall is in fact only in the mind of the pilot, it does not reach any bottom but is endless (Risset, 1985, p. 14).

Previous studies on the endless scale illusion have focused exclusively on the cognitive processes of pitch perception involved (Burns, 1981; Shepard, 1982; Shimizu et al., 2007). The tendency in these studies is to investigate the Shepard illusion by considering the perceptual system exclusively as a mechanism of feature extraction, at the same time discarding the possible affective properties of the illusion. The present study will adopt a different approach by focusing on the emotional connotations associated with the paradox. In particular, we will concentrate on the variant of the Shepard scale developed by composer and physicist Jean-Claude Risset, the Shepard-Risset glissando.

The Shepard-Risset glissando may give rise to a more complex subjective experience than has hitherto been known. When playing Risset's *Computer Suite from Little Boy* during university music courses, students often mentioned having experienced a disruption of equilibrium, eventually leading to the sensation of falling. Therefore, the aim of the present study was to investigate this phenomenon more systematically.

The analogy of music and motion dates back to Aristoxenus (Aristoxenus, *Harmonika Stoicheia*, trans. 1902, I:8–9; Rocconi, 2003; Hagel, 2010), fourth century BCE, and is reflected in our everyday language when we speak, for example, of “rising” and “falling” pitch. This metaphor has found confirmation in experimental studies, which have shown that pitch height is strongly associated with spatial verticality (Eitan and Granot, 2004). Additionally, Hedger et al. (2013) found that ascending and descending musical scales can elicit a visual motion aftereffect. They also suggest that the pitch-verticality coupling may result not only from cultural conditioning, but may be perceptually rooted in the stimulation of visual direction-selective neurons during listening to ascending and descending musical scales. Up to date the role of bodily feelings in emotion experience during music listening has been investigated only in relation to intensely pleasurable responses called “shivers” or “chills” (Blood and Zatorre, 2001; Salimpoor et al., 2009; Nusbaum and Silvia, 2011). Furthermore, while previous studies have investigated illusory self-motion sensation induced by moving auditory images (Väljamäe, 2009) and the effect of stationary auditory fields on postural balance (Sakellari and Soames, 1996), the relation between music listening and disturbance of equilibrium has not yet been examined.

Several studies suggest a link between vestibular processes, including perception of illusory motion, and negative emotion based upon overlapping neural networks (Balaban and Thayer, 2001; Carmona et al., 2009; Mast et al., 2014) and a shared right-hemisphere laterality (Carmona et al., 2009). One key region involved in vestibular/affective interactions is the parabrachial nucleus, linking the vestibular nuclei with limbic structures such as the amygdala, insula, and hypothalamus. Another possible underlying mechanism for the integration of vestibular information and emotion involves the interconnection between vestibular sensory areas and prefrontal regions. Further support for the relationship between movement perception and emotional response comes from studies linking direction of movement and emotional valence: Casasanto and Dijkstra (2010) as well as Zhang et al. (2015) have provided experimental evidence for the mental metaphor associating upward motion with positive and downward motion with negative emotional valence.

Furthermore, emotional response may be evoked by the Shepard-Risset glissando due to the ambiguous nature of its constituent tones. Ambiguity as a potential generator of emotion was first theorized by Meyer (1956). Meyer claimed emotional response arises from ambiguity inherent in musical patterns: musical compositions challenge our expectations by delaying or inhibiting their realization and, as a result, emotional response is generated. Similarly, Gombrich (1960) argued that knowledge influences our perception, and thus our experience, through the formation of expectations and hypothesis. Since then, musical expectancy has generally been considered a powerful mechanism for eliciting emotional response (Juslin and Västfjäll, 2008), involving distinct physiological systems: imagination, tension, prediction, reaction, and appraisal (Huron, 2006). Various studies have also provided empirical evidence for the role of expectancy in musical emotions (Sloboda, 1991; Koelsch, 2005; Steinbeis et al., 2006).

Although the debate about the precise nature of the relationship between music and emotions is still ongoing (Swaminathan and Schellenberg, 2015), previous self-reports and survey studies on the subjective experience of music have documented a wide range of emotional reactions: happiness, calmness, longing, tenderness, bliss, ecstasy, awe, wonder, but also sadness, melancholy, tension, and anxiety have been frequently reported by the participants (Juslin and Laukka, 2004; Zentner et al., 2008; Juslin, 2011). Interestingly, the more aesthetic emotional responses like “wonder,” “awe,” and “chills” seem to occur only rarely (Juslin et al., 2010). Furthermore, measures of the central nervous system as well as those of the peripheral nervous system have shown that music listening induces activity similar to that encountered with emotion processing in other domains. Both lesion studies and brain mapping studies suggest there is a distinct neural emotional pathway for music processing that is separate from those involved in music perception and memory (Peretz, 2010). Music induces a wide range of emotions that recruit subcortical and cortical structures similar to those involved in emotion processing, reward and arousal in other domains (Koelsch et al., 2010). Indeed, music-evoked emotions are associated with activation in both limbic and paralimbic brain structures.

Since interpersonal differences in personality may affect our affective response to music (Swaminathan and Schellenberg, 2015), particular attention will also be given to the relation between the emotional reactions to the Shepard-Risset illusion and personality. Indeed, studies have shown that personality may condition music preference (Rentfrow and Gosling, 2003; Garrido and Schubert, 2011; Ladinig and Schellenberg, 2012), the frequency of occurrence of specific musical emotions (Juslin et al., 2008; Barrett et al., 2010; Nusbaum and Silvia, 2011), the intensity of felt emotions (Kreutz et al., 2008; Vuoskoski et al., 2012; Liljeström et al., 2013), as well as the experience of chills (Grewe et al., 2007).

In the present study we used the Five-Factor Model to assess individuals' personality traits (Costa and McCrae, 1992; John and Srivastava, 1999). The Five-Factor Model, also called Big Five model of personality traits, is a widely used measure that delineates five broad personality dimensions: Extraversion, Agreeableness, Conscientiousness, Neuroticism, and Openness to Experience. The latter one in particular has shown to play an important role in our emotional reactions to music. Openness to Experience describes breadth, depth, originality, and complexity of an individual's mental and experiential life (John and Srivastava, 1999) and has been related to aesthetic sensitivity, creativity, curiosity, and imagination. Previous studies have associated this personality trait with the liking of complex and novel music and with the intensity of felt emotions. Indeed, both Vuoskoski et al. (2012) and Liljeström et al. (2013) reported that individuals scoring high on the trait Openness to experience experienced more intense emotions than listeners scoring low during listening to music. Similarly, Nusbaum and Silvia (2011) found that openness to experience was the only personality factor with a significant effect on the experience of the intense aesthetic response known as “chills.” In addition, Rentfrow and Gosling (2003) have shown that individuals scoring high on Openness to experience tend to enjoy listening to reflective and complex music (e.g., classical, jazz, blues, and folk), while extraversion, agreeableness, and conscientiousness are indicators for liking of upbeat and conventional music. Finally, in examining the individual frequency of occurrence of specific musical emotions Juslin et al. (2008) found correlations between prevalence of experienced emotions and the Big Five personality factors. In particular, pleasure-enjoyment correlated positively with neuroticism and negatively with openness to experience, while anxiety-fear correlated positively with conscientiousness. Correlations between personality and music perception observed in these studies were mostly small (*r* < 0.3) or, less often, medium (0.3 < *r* < 0.5).

In summary, the present study seeks to investigate the emotional experience elicited by the Shepard-Risset glissando, an auditory illusion frequently used in twentieth-century musical works. The subjectively experienced states were investigated with self-report measures concentrating both on musical emotions and on the experience of disturbance of equilibrium. We hypothesized that the Shepard-Risset glissando would elicit the sensation of disruption of equilibrium more frequently than an ordinary orchestral glissando and that this sensation would be associated with negative emotional responses. We also aimed to determine whether and how personality dimensions influence emotional response to musical illusions. In line with previous research, we expected “Openness to experience” to emerge as a salient personality dimension to predict heightened emotional response.

## Materials and methods

### Participants

Seventy-three volunteers (49 females and 24 males) participated in the present study (mean age 21.3 years; *SD* = 5.6). Subjects were recruited through advertisement and by word of mouth from Udine University population. None of the participants had professional musical expertise, 65 individuals reported to have some kind of practical and/or theoretic musical knowledge (52 of which at a basic level and 13 at an advanced level), while seven persons had no musical expertise at all. Written informed consent was obtained from each subject prior to participation in the experiment. The study was approved by the Ethics Committee of the University Hospital of Udine and was in accordance with the 1964 Declaration of Helsinki.

### Stimuli and measures

Three different musical stimuli containing glissandi were presented to the participants. The Shepard-Risset glissando was presented both within the aesthetic context of a musical composition and on its own. The musical excerpt of the illusion was taken from Claude Risset's *Computer Suite from Little Boy*, in which the second movement *Fall* (duration 2′50″) consists entirely of descending endless glissandi and scales (Risset, 1968/1988, track 5). The endless glissandi in this movement are partially superimposed with other shorter glissandi, a discrete Shepard scale and other brief sound events. The version of the Shepard-Risset glissando illusion not integrated in the aesthetic context of a musical composition consisted of a 90 s “raw” Shepard-Risset glissando generated using Matlab 2010b (min. freq. 27.5 Hz, 9 components, 12″/octave). Finally, the stimulus material also included an ordinary non-looping orchestral glissando. For this purpose, we used the opening 78 s of Iannis Xenakis's *Metastasis* (Xenakis, 1954/2001, track 3, 0–78″), which presents a long ascending orchestral glissando. Both musical excerpts were recorded from commercially available CDs. All stimuli were processed using Isotope RX2. Stimulus preparation included, where necessary, cutting and adding linear fade-in and fade-outs (500 ms) at the beginning and end of the excerpt. All auditory stimuli are available in the Supplementary Materials. The auditory stimuli were presented binaurally with AKG headphones (K271 mkII). The experiment was conducted using OpenSesame 2.9 software (Mathôt et al., 2012).

Subjects were asked to rate their emotions experienced during listening to the three musical stimuli on a 15-item, seven-point Likert scale. The adjectives used to describe the emotional response were: happy, tense, amazed, impatient, melancholic, meditative, joyful, agitated, nervous, irritated, sad, serene, relaxed, anxious, disturbed. Fourteen out of the fifteen adjectives were chosen from the list of 66 music-relevant emotion terms used in study 3 of Zentner et al. (2008). To these we then added “disturbed,” previously used in Grewe et al. (2010), as a possible indicator for psychological or physical disruption of equilibrium. All items had a seven-point response scale, with 0 indicating “not at all” and seven “very much.”

For each musical stimulus, subjects were also asked to indicate whether the piece of music had induced a disruption of equilibrium. If so, they were asked whether they also perceived a sensation of falling. Finally, to assess different domains of personality we used the Italian adaptation of the 44-item Big Five Inventory (BFI; Ubbiali et al., 2013). All items had a five-point response scale, ranging from “disagree strongly” to “agree strongly.” Together with the BFI all the subjects completed a brief questionnaire in which they were asked to report their gender, age, musical expertise, and listening habits.

### Procedure

The listening experiments were conducted individually on a computer (Apple iMac 8.1 with Windows XP) with headphones. Prior to beginning the study, all participants received instructions for the experiment. The instructions emphasized that answers to the questionnaires should only concern subjectively felt emotions, not the emotion expressed by the piece of music.

Participants were asked to listen attentively to the stimuli and keep their eyes closed during the presentation. Immediately after each stimulus ended, the Likert scale for emotion rating was presented on the screen. The scale was followed by the yes/no questions regarding disruption of equilibrium and the sensation of falling. Gravito-inertial disorientation is known to generate strong emotional reactions, such as fear and anxiety, because it presents a potential threat to the organism (Balaban and Thayer, 2001; Mast et al., 2014). As a result, disequilibrium is a highly disturbing experience that is unlikely to be forgotten in the short time it takes to fill out the emotion ratings.

Finally, participants were also asked to indicate whether they had liked the piece they had just heard.

After completing the listening experiment, subjects filled out the BFI. Ten individuals chose not to compile the BFI; therefore, our sample for all analyses concerning the personality test consisted of 63 subjects.

### Data analysis

The data were analyzed with Statistica 8 (StatSoft, Inc, Tulsa, OK). The matrix of the emotional response data obtained with the emotion ratings showed that 1.9% of the cells were left empty globally for the three stimuli. For data analysis, these empty cells were replaced with the mean values of the relevant item on the scale. The first aim of the present study was to investigate emotional responses elicited by the endless glissando illusion, presented both inside and outside of an aesthetic context (Risset Computer suite and Matlab Shepard-Risset Glissando, respectively), and by an ordinary glissando (Xenakis's *Metastasis*). The musical stimuli used in this experiment, albeit similar, differ from one another in a number of ways: while the Matlab glissando is unadorned and descending, the descending Risset Computer Suite glissando is partially superimposed with other shorter glissandi and sound events. Furthermore, contrary to the endless glissandi, the Xenakis glissando is both non-looping and ascending. In light of these differences we chose to analyze each of the stimuli separately.

First, repeated-measure ANOVAs separately tested the emotional response to each musical stimulus. The ANOVAs included Emotional Valence at two levels (Positive, Negative) as within-subject factor. Emotion categories were divided into positively and negatively valenced states following the partitioning generally found in emotion research (Russell, 1980; Trost et al., 2012). Separately for each musical stimulus, the data for positive valence emotions were obtained by averaging emotion ratings of the Likert scale for happy, meditative, joyful, serene, amazed, and relaxed emotions. Data for negative valence emotions were given by averaging emotion ratings of the Likert scale for tense, impatient, melancholic, agitated, nervous, irritated, sad, anxious, and disturbed emotions.

Next, we sought to find out if the stimuli can also evoke a disruption of equilibrium, and specifically if they can induce a feeling of falling. To this end, we first compared frequencies of disruption of equilibrium experiences, and associated feelings of falling, occurring within the three stimuli. A series of chi-square analyses was performed. Moreover, to assess potential differences in the emotional response given to the three musical stimuli between subjects who had and had not experienced a sensation of equilibrium disturbance, a mixed model ANOVA was carried out separately for each musical stimulus with Equilibrium disturbance (Yes, No) as between-subject factor and Emotional Valence (Positive, Negative) as within-subject variable.

Finally, since previous studies have shown that various personality dimensions can influence the subjective response to music, we tested for possible associations between personality traits and emotional responses and feelings of equilibrium disturbance within the three types of glissandi. First, a series of parametric correlations were run between BFI dimensions and positive and negative emotional valence associated with each of the three musical stimuli. Second, a series of independent-sample *t*-tests for the five BFI dimensions were performed in order to investigate whether there were personality differences between the subjects who experienced a sensation of disturbance of equilibrium vs. the individuals who did not have such an experience while listening to the three musical stimuli.

A 0.05 significance threshold was used in all statistical tests. In all ANOVAs, significant interactions were followed-up with Bonferroni's *post hoc* tests. In the analyses, effect sizes are reported as partial eta squared (η_*p*_^2^). Effect sizes for the chi-square tests are reported as Φ.

## Results

### Emotional response to the musical stimuli

**Figure 1:**
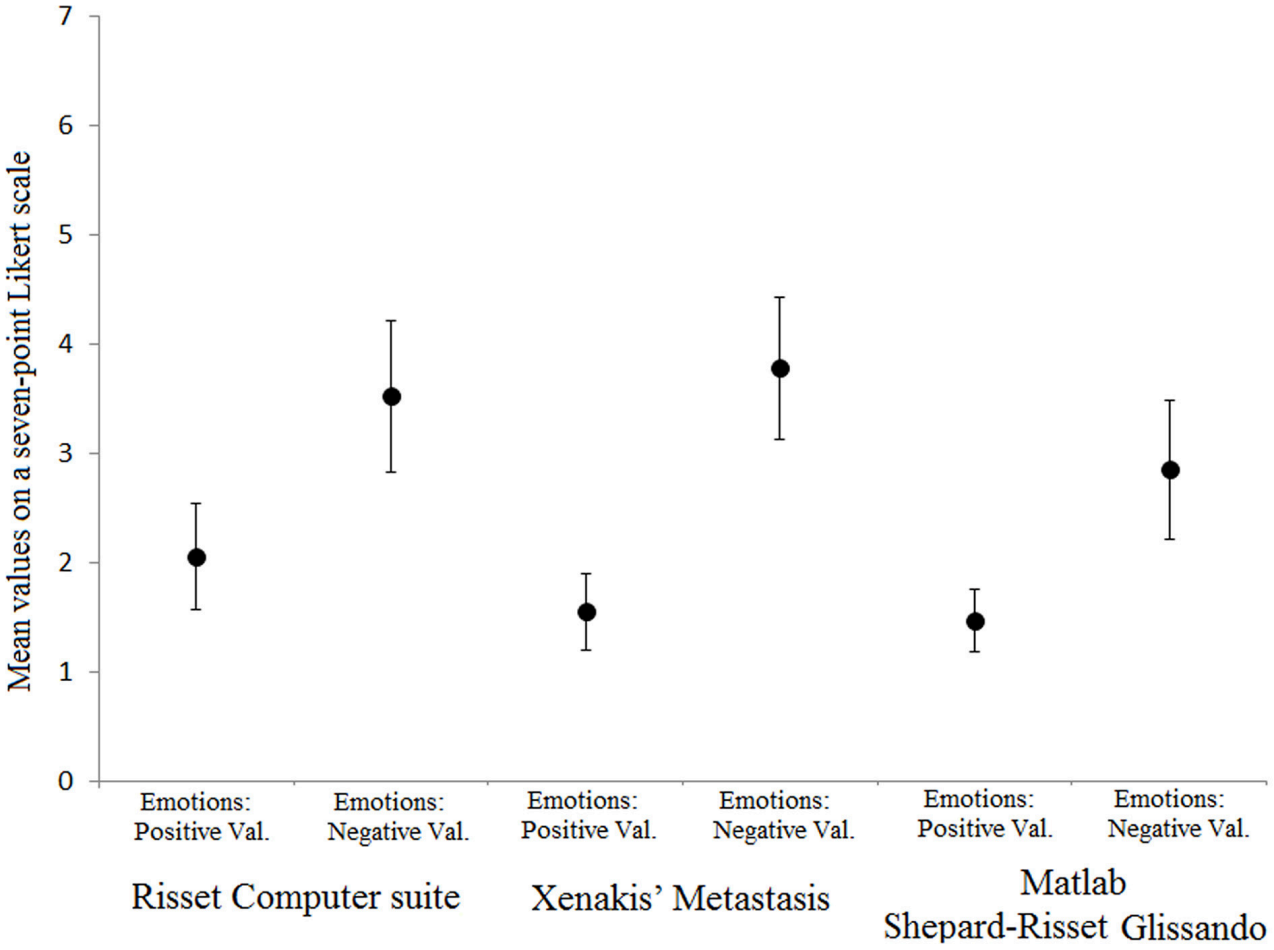
**Mean values for emotions with positive and negative valence for each musical stimulus**. Values are based on judgments given on a seven-point scale. Error bars represent standard deviations of the means.

The mean ratings of positive and negative emotional valence are reported for each musical stimulus in Figure 1 (see Table 1S for the ratings referring separately to all 15 emotions). The ANOVAs carried out separately for each musical stimulus (Risset Computer suite, Xenakis's *Metastasis*, Matlab Shepard-Risset Glissando) on the positive and negative emotion ratings showed the main effect of Emotional Valence for each stimulus (Table 1). The results indicated higher scores for negative emotional valence than for positive emotional valence for each stimulus. In other words, for each musical stimulus negative emotions were generally rated higher than positive emotions and this is in line with the percentage of participants responding “no” to the debriefing question “Did you like the music?” asked after listening of each stimulus. This percentage was 68.5% for Risset Computer suite, 67.2% for Xenakis's *Metastasis*, and 89.1% for Matlab Shepard-Risset Glissando.

**Table 1:** **Anova results for analyses on emotional response and disruption of equilibrium associated with the three musical stimuli (Risset Computer suite, Xenakis's *Metastasis*, Matlab Shepard-Risset Glissando): *F*-values, *p*-values, and Effect Sizes (η_*p*_^2^) for each type of analysis**.

**Type of analysis**		**Risset computer suite**	**Xenakis's *Metastasis***	**Matlab Shepard-Risset glissando**
**EMOTIONAL RESPONSE TO THE MUSICAL STIMULI**				
Positive and negative emotions	Main effect of emotional valence	*F*_(1, 72)_ = 42.36, *p* < 0.001; *η_*p*_*^2^ = 0.370	*F*_(1, 72)_ = 160.37, *p* < 0.001; *η_*p*_*^2^ = 0.690	*F*_(1, 72)_ = 61.62, *p* < 0.001; *η_*p*_*^2^ = 0.461
**GLISSANDO ILLUSIONS AND DISRUPTION OF EQUILIBRIUM**				
Positive and negative emotions	Main effect of emotional valence	*F*_(1, 71)_ = 46.98, *p* < 0.001; *η_*p*_*^2^ = 0.398	*F*_(1, 71)_ = 149.69, *p* < 0.001; *η_*p*_*^2^ = 0.678	*F*_(1, 71)_ = 70.39, *p* < 0.001; *η_*p*_*^2^ = 0.498
	Main effect of equilibrium disturbance	*F*_(1, 71)_ = 2.05, *p* = 0.156; *η_*p*_*^2^ = 0.028	*F*_(1, 71)_ = 0.72, *p* = 0.398; *η_*p*_*^2^ = 0.010	*F*_(1, 71)_ = 4.81, *p* = 0.031; *η_*p*_*^2^ = 0.063
	Two-way interaction	*F*_(1, 71)_ = 12.40, *p* < 0.001; *η_*p*_*^2^ = 0.149	*F*_(1, 71)_ = 0.98, *p* = 0.326; *η_*p*_*^2^ = 0.013	*F*_(1, 71)_ = 7.99, *p* = 0.006; *η_*p*_*^2^ = 0.101

### Glissando illusions and disruption of equilibrium

We tested whether the three glissando stimuli could differentially evoke a feeling of disruption of equilibrium eventually leading to the sensation of falling. A different number of participants reported a feeling of disturbance of equilibrium depending on the listened musical stimulus. In particular, a larger number of participants reported such a feeling after listening to the Risset Computer suite (38/73 participants) than the Xenakis (24/73) or the Matlab Shepard-Risset Glissando (16/73). These differences were significant: $\chi_{\left(1\right)}^{2}$ = 4.74, *p* = 0.029, Φ = 0.192 and $\chi_{\left(1\right)}^{2}$ = 12.96, *p* < 0.001, Φ = 0.311, respectively, (Yates correction applied) [$\chi_{\left(1\right)}^{2}$ = 1.69, *p* = 0.194, Φ = 0.122 for Xenakis's *Metastasis* vs. Matlab Shepard-Risset Glissando]. On average for the three stimuli, 87.6% of the subjects who had a sensation of disruption of equilibrium reported such sensation as unpleasant rather than pleasant.

Turning to the connected sensation of falling, which was investigated for those subjects reporting disturbance of equilibrium, we found that 21/38 participants said to have experienced a sensation of falling while listening to the Risset Computer suite vs. 3/24 for Xenakis's *Metastasis* [$\chi_{\left(1\right)}^{2}$ = 9.61, *p* = 0.002, Φ = 0.426] and 14/16 for the Matlab Shepard-Risset Glissando [$\chi_{\left(1\right)}^{2}$ = 3.81, *p* = 0.051, Φ = 0.308] [$\chi_{\left(1\right)}^{2}$ = 19.13, *p* < 0.001, Φ = 0.742 for Xenakis's *Metastasis* vs. Matlab Shepard-Risset Glissando]. Thus, the Risset Computer suite stimulus appeared to be particularly capable of eliciting feelings of disruption of equilibrium; moreover, both Shepard-Risset glissando stimuli, independently of whether the illusion was employed within the aesthetic context of a composition, also frequently led to the associated feeling of falling, much more often than the stimulus containing an ordinary orchestral glissando.

**Figure 2:**
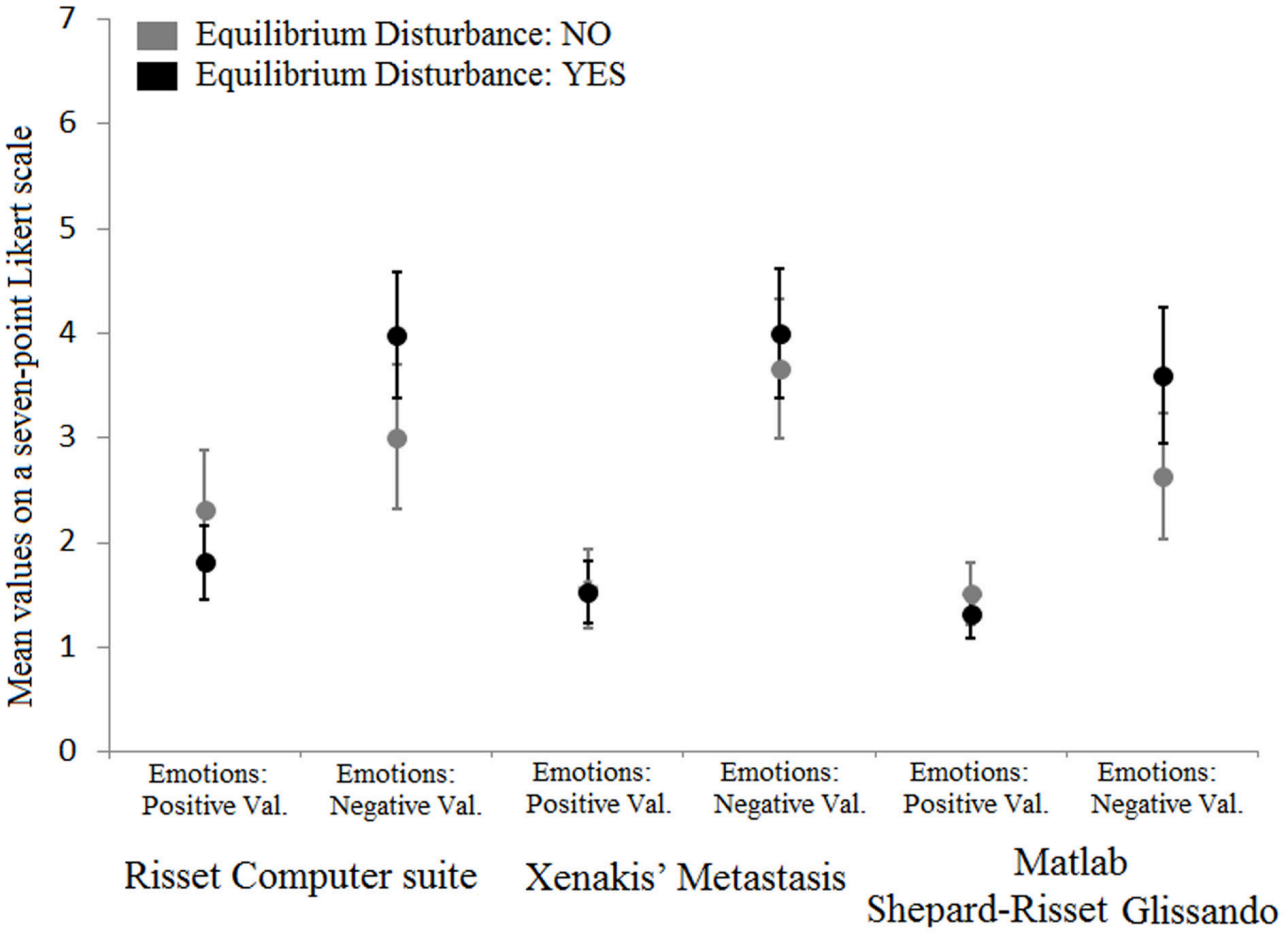
**Mean values for emotions with positive and negative valence for each musical stimulus and for both subjects who experienced and did not experience a feeling of equilibrium disturbance while listening to the stimuli**. Error bars represent standard deviations of the means.

Next, we assessed possible differences in emotional response given to the three musical stimuli between subjects who had and had not experienced a sensation of disturbance of equilibrium while listening to the stimuli. The 2 (Equilibrium disturbance: Yes, No) × 2 (Emotional Valence: Positive, Negative) ANOVA carried out on the emotion ratings of the Likert scale relative to the Risset Computer suite stimulus showed the main effect of Emotional Valence but not that of Equilibrium disturbance; however, the two-way interaction was significant (Table 1). *Post-hoc* tests carried out to analyze this two-way interaction revealed higher scores for negative emotional valence among subjects who had experienced a feeling of disturbance of equilibrium relative to those who had not had the same experience (*p* = 0.002; Figure 2). No difference between subjects was found for positive emotional valence (*p* = 0.354).

Furthermore, a corresponding 2 (Equilibrium disturbance: Yes, No) × 2 (Emotional Valence: Positive, Negative) ANOVA was carried out on the emotion ratings of the Likert scale relative to the Xenakis Metastasis stimulus. The analysis only showed the main effect of Emotional Valence (Negative > Positive) but not that of Equilibrium disturbance or the two-way interaction (Figure 2 and Table 1).

Finally, a 2 (Equilibrium disturbance: Yes, No) × 2 (Emotional Valence: Positive, Negative) ANOVA was carried out on the emotion ratings of the Likert scale relative to the Matlab Shepard-Risset Glissando stimulus. Similarly to the Risset Computer suite stimulus, the analysis showed the main effects of Emotional Valence and Equilibrium disturbance and also the two-way interaction (Table 1). *Post-hoc* tests revealed higher scores for negative emotional valence for subjects who had vs. had not, experienced a feeling of disturbance of equilibrium (*p* = 0.003; Figure 2). No difference was found for positive emotional valence (*p* > 0.99).

To sum up, differences in emotional responses were observed between subjects who had and had not experienced a sensation of disturbance of equilibrium for both Shepard-Risset glissando stimuli (Risset Computer suite stimulus and Matlab Shepard-Risset Glissando stimulus) but not for the Xenakis Metastasis stimulus. In particular, such differences were expressed in terms of higher negative emotional ratings given by those who had experienced a feeling of disturbance of equilibrium during listening to stimuli (see Table 1S for the ratings referring separately to all 15 emotions).

### Glissando illusions: association with personality dimensions

Finally, we investigated possible associations between personality traits as measured by the BFI and emotional responses to glissando illusions. First of all, scale scores were obtained for the 63 individuals who undertook the BFI by averaging the items for each BFI personality trait. Scale scores were: *M* = 3.11 (*SD* = 0.79) for Extraversion, *M* = 3.65 (*SD* = 0.51) for Agreeableness, *M* = 3.45 (*SD* = 0.73) for Conscientiousness, *M* = 3.35 (*SD* = 0.71) for Neuroticism, and *M* = 3.86 (*SD* = 0.64) for Openness to Experience. These values were totally in line with those of the normative sample of Ubbiali et al. (2013).

Moreover, we ran correlations separately for the three stimuli between BFI personality dimensions and positive and negative emotional responses. After adjusting α to 0.0017 (i.e., a Bonferroni correction for 3*2*5 comparisons was applied) to control for an inflated type I error, the only significant correlations concerned the agreeableness trait, which negatively correlated with positive emotion ratings of the Matlab Shepard-Risset Glissando stimulus (*r* = −0.41, *p* = 0.001), and the openness to experience dimension, which positively correlated with positive emotion ratings of the Risset Computer suite stimulus (*r* = 0.40, *p* = 0.001).

Finally, in order to investigate whether there are personality differences between the subjects who experienced a sensation of disturbance of equilibrium vs. the individuals who did not have such an experience while listening to the three musical stimuli, we ran a series of independent-sample *t*-tests for the 5 BFI dimensions and separately for the three musical stimuli. The *t*-tests only showed an effect for the neuroticism factor in the Risset Computer suite stimulus [*t*_(61)_ = −2.05, *p* = 0.044, Cohen's *d* = 0.53; all other *t*_(61)_ < 1.97, *p* > 0.05] indicating higher neuroticism for those who experienced an equilibrium disturbance (*n* = 33; *M* = 3.52, *SD* = 0.68) relative to subjects who did not have this experience (*n* = 30; *M* = 3.16, *SD* = 0.71; see Table 1S for the mean values concerning all other BFI dimensions and musical stimuli). Although the relative size of Cohen's *d* indicates a medium effect, it should be noted that such effect would not survive a strict Bonferroni correction for the number (5*3) of tested comparisons. This calls for caution when interpreting the significance of this result.

## Discussion

The purpose of this research was to investigate the emotional experience induced by listening to the Shepard-Risset glissando illusion and to an ordinary glissando. Both common emotional responses, like happy, sad, relaxed, anxious, or meditative, as well as disruption of equilibrium were examined. The results were then viewed in light of the five personality traits measured by the Five-factor model of personality traits.

### Emotional experience and disruption of equilibrium

Our study provided novel insights into the relation between music listening and disturbance of equilibrium. The study confirmed our hypothesis that the endless scale illusion in Risset's *Computer Suite* is particularly capable of evoking a disruption of equilibrium. Indeed, frequency of occurrence of this sensation was significantly higher for the Risset excerpt than for the other two stimuli. This sensation is generally judged unpleasant by the participants. The Shepard-Risset glissando also appeared to elicit the associated feeling of falling much more often than the ordinary orchestral glissando, independent of whether the musical paradox was incorporated into a musical composition or not. However, we must be cautious when attributing these differences solely to the paradoxical aspect of the Shepard-Risset glissando. As already mentioned in the methods, while the Shepard-Risset glissando was descending, Xenakis's glissando was ascending. The rising motion of the latter may have contributed to its inaptness for eliciting the falling sensation.

To the best of our knowledge our study is the first to show that musical stimuli can provoke a disturbance of equilibrium. Destabilizing effects of sound have previously been reported in relation to acoustic stimuli such as clicks or pure tones (Sakellari and Soames, 1996; Forti et al., 2010), but not for musical stimuli. Instead, music listening has been shown to improve balance maintenance. Forti et al. (2010), for example, found that some types of music may increase accuracy of postural control. Our experiment shows that listening to a descending Shepard-Risset glissando illusion, both within the aesthetical context of a composition and on its own, can elicit bodily feelings resulting in the sensation of loss of equilibrium, and in particular of falling.

The fact that the musical paradox did not elicit disruption of equilibrium in all participants is consistent with past work on physiological phenomena in response to music listening. Previous studies on chills, or “shivers-down-the spine,” showed that some people have never experienced this intensely pleasurable response and that only a small percentage of participants report chills while listening to experimenter-chosen stimuli during lab sessions (Blood and Zatorre, 2001; Grewe et al., 2007; Nusbaum and Silvia, 2011). Therefore, our findings extend the results of Nusbaum and Silvia (2011) to intensely unpleasurable responses. Nonetheless, it cannot be ruled out that the currents findings showing an influence of music on disturbance of equilibrium may be due, at least in part, to a demand effect in that participants might have associated disequilibrium with the stimuli rather than having actually experienced it. Future studies may continue to use self-report measures as well as more objective indexes of the effects of music on balance, for instance by measuring stabilometric variables such as frequency and amplitude of body sway during music listening (e.g., Väljamäe, 2009; Forti et al., 2010).

Our main results concerning the patterns of emotional response to the three stimuli used (Risset's *Computer suite for Little Boy*, Xenakis's *Metastasis*, Matlab Shepard-Risset Glissando) showed that in all three stimuli the participants rated the negatively valenced emotions (i.e., anxious, impatient, disturbed, irritated, melancholic, nervous, tense, sad) higher than the positively valenced ones (i.e., happy, joyful, meditative, amazed, relaxed, serene).

The negative pleasure ratings of the pieces observed in the experiment (in all three stimuli less than a third of the participants liked the musical piece) are in line with previous studies if we consider the wide range of high arousing negative states measured in the emotion ratings (anxious, agitated, disturbed, impatient, irritated, nervous). Indeed, Vuoskoski et al. (2012) showed that unlike low-arousing sadness or melancholic states, we do not enjoy the high arousing “negative” states, like anxiety or fear when listening to music.

Moreover, differences in emotional responses were observed between subjects who had and had not experienced the sensation of disruption of equilibrium while listening to the Risset and Matlab stimuli. Specifically, disruption of equilibrium was accompanied by higher negative emotion ratings. Instead, no significant differences in emotional response were observed for the Xenakis stimulus.

The present findings suggest that Shepard-Risset's musical paradox holds important emotional connotations. The experienced emotions to the illusion may be mediated by different underlying psychological mechanisms. Juslin and Västfjäll (2008) identified six psychological mechanisms through which music might arouse emotions: brain stem reflexes, evaluative conditioning, emotional contagion, visual imagery, episodic memory, and musical expectancy. Although the psychological mechanisms were not directly investigated in our experiment, we hypothesize that musical expectancy and visual imagery may have mediated the listener's response to the endless glissando illusion. Musical expectancy refers to a process whereby emotions arise from the arousal and suspension or fulfillment of expectations. The arousal and suspension of expectations may lead to feelings of tension and anxiety (Meyer, 1956), which, according to Steinbeis et al. (2006), predispose the listener to an increase in overall emotionality. The ambiguous nature of the musical tones used in the Shepard-Risset glissando, together with the paradoxical effect of a glissando that descends endlessly but never really seems to get any lower in pitch creates a highly unusual, and upsetting, course of events which in turn leads to a state of uncertainty in which our expectations are constantly unfulfilled. In this framework, our results may lend support for the notion of ambiguity in music as a powerful trigger for emotional response. However, for the emotional response to be aesthetically meaningful, the tension must be followed by a release (Meyer, 1956). Hence, the absence of a resolution in the Shepard-Risset glissando may explain the negative pleasure ratings observed in the experiment.

Emotional response and the disruption of equilibrium in particular, may be further mediated by visual imagery. Visual imagery occurs when the emotional response is mediated by mental images evoked during music listening. Juslin and Västfjäll (2008) hypothesize that listeners conceptualize the musical structure through a metaphorical nonverbal mapping between the music and so-called image-schemata grounded in bodily experience. As discussed above in the introduction, decreasing musical pitch can conjure up mental images of downward motion, like falling. Previous studies have shown that visual, auditory, and motor imagery are processed in the same way as their real-world counterparts (Kleber et al., 2007; Juslin and Västfjäll, 2008; Mast et al., 2014) and can thus similarly trigger an emotional response.

### The role of personality

We investigated whether interpersonal differences in personality could account for the variability in emotional response to the glissando stimuli. We found that “Openness to experience,” a trait reflecting dispositional curiosity and creativity, correlated positively with positive emotions induced by the Risset Computer Suite. Moreover, the agreeableness trait, a trait expressing individuals' tendency for compassion and empathy, correlated negatively with positive emotion rating for the Matlab stimulus. Finally, the individuals who experienced an equilibrium disturbance during listening to the Computer Suite showed higher neuroticism, with a medium effect size, than subjects who did not have this experience.

Overall, these data can be seen to complement the results of previous studies also showing complex patterns of personality/listening to music correlations (Juslin et al., 2008; Nusbaum and Silvia, 2011). As expected, Openness of experience emerged as a predictor for heightened positive emotions, albeit only in relation to the Risset Computer Suite. Openness to experience has been known to correlate with overall emotion intensity (Vuoskoski et al., 2012; Liljeström et al., 2013), but also with prevalence and intensity of positive emotions (Juslin et al., 2008; Liljeström et al., 2013), in particular in relation to negative-affect stimuli (Vuoskoski et al., 2012). Individuals with higher scores on the Openness dimension have increased aesthetic sensitivity and are open to novel experiences. Since individuals high in Openness are believed to be more tolerant of ambiguity (McCrae, 2007), this may explain why they experienced more positive emotions while listening to the Computer Suite.

More surprising was the relationship between agreeableness and emotion rating for the Matlab stimulus, since the Agreeableness trait has only rarely been associated with music listening. Liljeström et al. (2013) found that this trait correlated positively with positive musical emotions and negatively with negative emotions, but the authors call for further research to explain these relationships. Since in our experiment the relation between agreeableness and positive emotion ratings was found for the Matlab stimulus only, the question naturally arises as to whether the listeners perceived the Matlab-generated glissando as a musical excerpt or merely as an acoustical, non-aesthetic, stimulus. For nonmusical emotions, Juslin et al. (2008) observed a positive correlation between agreeableness and interest-expectancy. However, since agreeableness is generally associated with emotional processes that have consequences for interpersonal relationships (Tobin et al., 2000), its precise relationship with emotional response in our experiment remains unclear.

Finally, the uncorrected results on the association between personality traits and equilibrium disruption may indicate a relation between neuroticism and the experience of disruption of equilibrium while listening to the glissando illusion. Participants reporting having experienced disturbance of equilibrium while listening to Risset's *Computer Suite* had higher scores in the neuroticism dimension than individuals who had not experienced this state. Neuroticism has been associated with negative-affect stimuli in previous studies, but only rarely in relation to music. For example, Larsen and Ketelaar (1991) found that subjects with high scores in the neuroticism dimension showed heightened emotional reactivity to negative-affect stimuli. Although the observed result awaits replication, our findings seem to extend these earlier results into the domain of musical emotions. From another perspective, it was recently reported an association between equilibrium disturbance (i.e., benign paroxysmal positional vertigo or chronic subjective dizziness) and psychiatric symptomatology characteristic of neurotic personality such as anxiety, social dysfunction, and depression problems (Hagr, 2009; Staab et al., 2014). However, since the relation between high trait neuroticism and disruption of equilibrium was not observed for all three stimuli, but for the Risset stimulus only, further investigation is needed to reveal how personality factors and features of the Risset Computer suite jointly affect the experience of disequilibrium.

In short, our study provides insight into the emotional response to one musical paradox. The Shepard-Risset glissando shows that auditory illusions can hold an interest not only for the cognitive processes involved, but also for the emotional experience they elicit. This is particularly clear in Risset's *Computer Suite from Little Boy*. In the second section of this composition, appropriately entitled “Fall,” Risset exploits the possibilities of digital sound synthesis to elicit, in the listener, genuine emotional responses. The endless glissando illusion is used to convey the feeling of falling the pilot experiences when, in his dream, he identifies himself with the atomic bomb he dropped on Hiroshima. The fall experienced by the pilot occurs, however, entirely in his mind and, unlike that of the atomic bomb, does not reach any bottom but continues endlessly. Our results show that the infinitely descending pitch of the Shepard-Risset glissando elicits an emotional response such that listeners, just like the pilot portrayed by Risset in the *Computer Suite*, experience a feeling of equilibrium disturbance and of falling. In light of the emotional implications associated with the glissando illusion, it is of no surprise that this psychoacoustic effect has been incorporated in several musical works of the twentieth-century.

## Author contributions

All authors listed, have made substantial, direct and intellectual contribution to the work, and approved it for publication.

### Conflict of interest statement

The authors declare that the research was conducted in the absence of any commercial or financial relationships that could be construed as a potential conflict of interest.
