# Supplementary material for: Listening to the Shepard-Risset Glissando: the Relationship between Emotional Response, Disruption of Equilibrium, and Personality
Source: Front Psychol. 2016 Mar 4;7:300. doi: 10.3389/fpsyg.2016.00300 (PMC4777920; doi:10.3389/fpsyg.2016.00300)
Supplement: Supplementary file 1 [file Table1.DOCX]

Supplementary Material

**Listening to the Shepard-Risset glissando: the relationship between emotional response, disruption of equilibrium and personality**

**Eveline Vernooij^1^*, Angelo Orcalli^1^, Franco Fabbro^2,3^, Cristiano Crescentini^2^**

^1^Music and Audio Research Laboratories, Department of Human Sciences, University of Udine, Udine, Italy

^2^ Department of Human Sciences, University of Udine, Udine, Italy

^3^ Perceptual Robotics (PERCRO) Laboratory, Scuola Superiore Sant’Anna, Pisa, Italy

*** Correspondence:** Eveline Vernooij, Department of Human Sciences, University of Udine, Italy. email: [eveline.vernooij@uniud.it](mailto:eveline.vernooij@uniud.it)

**Table 1S**: Descriptive statistics of the emotional response to glissando musical stimuli and their associations with equilibrium disturbance and personality traits.

| *1. Emotional response to the musical stimuli* | | | | | | | | | | | | | | | | | | | | | | | | | | | | | | | | | | | |
| --- | --- | --- | --- | --- | --- | --- | --- | --- | --- | --- | --- | --- | --- | --- | --- | --- | --- | --- | --- | --- | --- | --- | --- | --- | --- | --- | --- | --- | --- | --- | --- | --- | --- | --- | --- |
| Music Stim | | | Pos Val  Emot | | Neg Val  Emot | | | |  | | | | | | | | | | | | | | | | | | | | | | | | | | |
| 1 | | | 2.05  (.96) | | 3.52  (1.38) | | | |  |  |  |  |  |  |  |  |  |  |  |  |  |  |  |  |  |  |  |  |  |  |  |  |  |  |  |
| 2 | | | 1.55  (.70) | | 3.78  (1.30) | | | |  |  |  |  |  |  |  |  |  |  |  |  |  |  |  |  |  |  |  |  |  |  |  |  |  |  |  |
| 3 | | | 1.46  (.57) | | 2.84  (1.28) | | | |  |  |  |  |  |  |  |  |  |  |  |  |  |  |  |  |  |  |  |  |  |  |  |  |  |  |  |
|  | | | | | | | | | | | | | | | | | | | | | | | | | | | | | | | | | | | |
| Music Stim | | | Pos Val Emot | | | | | | | | | | | | | | | | Neg Val Emot | | | | | | | | | | | | | | | | |
|  |  |  | HAP | | JOY | | | MED | | | AMA | | | REL | | | SER | | AGI | | | ANX | | IMP | | DIS | | IRR | | MEL | | NER | TEN | | SAD |
| 1 | | | 1.71  (1.04) | | 1.41  (.94) | | | 3.14  (1.77) | | | 2.57  (1.53) | | | 1.79  (1.29) | | | 1.68  (1.14) | | 3.76  (1.95) | | | 3.94  (1.94) | | 3.89  (1.84) | | 4.17  (1.83) | | 2.93  (1.67) | | 2.64  (1.65) | | 3.94  (2.06) | 4.31  (1.74) | | 2.06  (1.45) |
| 2 | | | 1.33  (.80) | | 1.19  (.49) | | | 1.86  (1.34) | | | 2.29  (1.58) | | | 1.29  (.68) | | | 1.33  (.71) | | 4.70  (1.93) | | | 4.77  (1.90) | | 3.86  (2.06) | | 4.89  (1.85) | | 2.89  (1.77) | | 1.89  (1.45) | | 4.24  (1.89) | 5.03  (1.76) | | 1.73  (1.34) |
| 3 | | | 1.27  (.58) | | 1.08  (.28) | | | 1.97  (1.29) | | | 1.63  (1.22) | | | 1.59  (1.14) | | | 1.25  (.74) | | 3.06  (1.85) | | | 3.13  (1.83) | | 3.40  (1.84) | | 3.21  (1.94) | | 3.42  (2.07) | | 1.74  (1.32) | | 3.17  (1.72) | 2.87  (1.62) | | 1.61  (1.14) |
| *2. Glissando illusions and disruption of equilibrium* | | | | | | | | | | | | | | | | | | | | | | | | | | | | | | | | | | | |
| Music Stim | Equ Dist:YES | | Pos Val  Emot | | Neg Val  emot | | | | Music Stim | | | Equ Dist: NO | | | Pos Val  Emot | | | Neg Val  Emot | | |  | | | | | | | | | | | | | | |
| 1 | N = 38/73 | | 1.81  (.72) | | 3.98  (1.21) | | | | 1 | | | N = 35/73 | | | 2.31  (1.13) | | | 3.01  (1.39) | | |  |  |  |  |  |  |  |  |  |  |  |  |  |  |  |
| 2 | N = 24/73 | | 1.53  (.61) | | 4.00  (1.24) | | | | 2 | | | N = 49/73 | | | 1.56  (.74) | | | 3.66  (1.33) | | |  |  |  |  |  |  |  |  |  |  |  |  |  |  |  |
| 3 | N = 16/73 | | 1.32  (.47) | | 3.60  (1.31) | | | | 3 | | | N = 57/73 | | | 1.50  (.59) | | | 2.63  (1.20) | | |  |  |  |  |  |  |  |  |  |  |  |  |  |  |  |
|  | | | | | | | | | | | | | | | | | | | | | | | | | | | | | | | | | | | |
|  | | | Pos Val Emot | | | | | | | | | | | | | | | | Neg Val Emot | | | | | | | | | | | | | | | | |
| Music Stim | Equ Dist:YES | | HAP | | JOY | | | MED | | | AMA | | | REL | | | SER | | AGI | | | ANX | | IMP | | DIS | | IRR | | MEL | | NER | TEN | | SAD |
| 1 | N = 38/73 | | 1.47  (.72) | | 1.25  (.49) | | | 2.79  (1.63) | | | 2.57  (1.57) | | | 1.44  (1.00) | | | 1.33  (.81) | | 4.49  (1.54) | | | 4.47  (1.77) | | 4.13  (1.77) | | 4.50  (1.54) | | 3.52  (1.78) | | 3.02  (1.73) | | 4.55  (1.87) | 4.90  (1.50) | | 2.24  (1.50) |
| 2 | N = 24/73 | | 1.21  (.66) | | 1.13  (.45) | | | 2.00  (1.47) | | | 2.33  (1.74) | | | 1.25  (.44) | | | 1.25  (.53) | | 5.17  (1.58) | | | 5.04  (1.92) | | 3.54  (2.04) | | 5.25  (1.73) | | 3.21  (1.84) | | 2.08  (1.56) | | 4.79  (1.59) | 5.17  (1.46) | | 1.79  (1.35) |
| 3 | N = 16/73 | | 1.19  (.41) | | 1.13  (.35) | | | 1.69  (.96) | | | 1.44  (.74) | | | 1.25  (.46) | | | 1.25  (.46) | | 4.25  (1.85) | | | 4.50  (1.88) | | 4.06  (2.03) | | 4.31  (1.77) | | 3.69  (2.20) | | 1.88  (1.10) | | 4.06  (1.83) | 3.88  (1.91) | | 1.75  (.94) |
|  | | | Pos Val Emot | | | | | | | | | | | | | | | | Neg Val Emot | | | | | | | | | | | | | | | | |
| Music Stim | Equ Dist:NO | | HAP | | JOY | | | MED | | | AMA | | | REL | | | SER | | AGI | | | ANX | | IMP | | DIS | | IRR | | MEL | | NER | TEN | | SAD |
| 1 | N = 35/73 | | 1.98  (1.26) | | 1.58  (1.24) | | | 3.52  (1.85) | | | 2.58  (1.52) | | | 2.17  (1.47) | | | 2.05  (1.33) | | 2.96  (2.06) | | | 3.37  (1.99) | | 3.62  (1.89) | | 3.80  (2.07) | | 2.28  (1.27) | | 2.24  (1.47) | | 3.28  (2.08) | 3.68  (1.77) | | 1.86  (1.40) |
| 2 | N = 49/73 | | 1.39  (.86) | | 1.22  (.51) | | | 1.80  (1.27) | | | 2.27  (1.52) | | | 1.31  (.77) | | | 1.37  (.78) | | 4.47  (2.06) | | | 4.63  (1.90) | | 4.02  (2.08) | | 4.71  (1.89) | | 2.73  (1.73) | | 1.80  (1.40) | | 3.96  (1.98) | 4.96  (1.89) | | 1.69  (1.34) |
| 3 | N = 57/73 | | 1.29  (.62) | | 1.07  (.26) | | | 2.05  (1.37) | | | 1.68  (1.32) | | | 1.69  (1.25) | | | 1.25  (.81) | | 2.72  (1.68) | | | 2.74  (1.62) | | 3.21  (1.78) | | 2.90  (1.84) | | 3.34  (2.04) | | 1.70  (1.39) | | 2.92  (1.58) | 2.59  (1.40) | | 1.57  (1.19) |
| *3. Glissando illusions: association with personality dimensions* | | | | | | | | | | | | | | | | | | | | | | | | | | | | | | | | | | | |
| Music  Stim | | Equ Dist: YES | | EXT | | AGR | CON | | | NEU | | | OPE | | | Music  Stim | | | | Equ Dist: NO | | | EXT | | AGR | | CON | | NEU | | OPE | | |  | |
| 1 | | 30/63 | | 3.24  (.75) | | 3.70  (.51) | 3.43  (.75) | | | 3.52  (.68) | | | 3.80  (.71) | | | 1 | | | | 33/63 | | | 2.96  (.83) | | 3.60  (.52) | | 3.47  (.73) | | 3.16  (.71) | | 3.94  (.57) | | |  |  |
| 2 | | 19/63 | | 3.39  (.72) | | 3.53  (.50) | 3.18  (.68) | | | 3.43  (.64) | | | 3.91  (.74) | | | 2 | | | | 44/63 | | | 2.99  (.80) | | 3.70  (.52) | | 3.57  (.73) | | 3.32  (.75) | | 3.85  (.61) | | |  |  |
| 3 | | 14/63 | | 3.13  (.86) | | 3.59  (.54) | 3.52  (.62) | | | 3.60  (.60) | | | 3.76  (.74) | | | 3 | | | | 49/63 | | | 3.10  (.79) | | 3.67  (.51) | | 3.43  (.77) | | 3.28  (.73) | | 3.89  (.63) | | |  |  |

Notes: For parts 1 and 2 of the table, mean values (and standard deviations of the means in parentheses) are based on emotion ratings given on a seven-point Likert scale for each musical stimulus. For part 3 of the table, mean values (and standard deviations of the means in parentheses) refer to scores obtained in the Big Five model of personality traits (EXT= Extraversion; AGR= Agreeableness; CON= Conscientiousness; NEU= Neuroticism; OPE= Openness to Experience). Music Stim 1 = Risset Computer suite; Music Stim 2 = Xenakis’s Metastasis; Music Stim 3 = Matlab Shepard-Risset Glissando. Equ Dist: YES = (number of) participants reporting a feeling of disturbance of equilibrium during listening to musical stimuli; Equ Dist: No = (number of) participants not reporting a feeling of disturbance of equilibrium during listening to musical stimuli. Pos Val Emot = positive emotional valence; Neg Val Emot = negative emotional valence. Positive emotional valence includes: HAP = happy; JOY = joyful; MED = meditative; AMA = amazed; REL = relaxed; and SER = serene. Negative emotional valence includes: AGI = agitated; ANX = anxious; IMP = impatient; DIS = disturbed; IRR = irritated; MEL = melancholic; NER = nervous; TEN = tense; and SAD = sad.
